# Bilateral optic neuropathy following vincristine chemotherapy: A case report with description of multimodal imaging findings

**DOI:** 10.1097/MD.0000000000024706

**Published:** 2021-03-05

**Authors:** Woo Hyuk Lee, Sun Kyoung You, Yeon-Hee Lee

**Affiliations:** a Department of Ophthalmology, Gyeongsang National University Changwon Hospital, Changwon; b Department of Radiology; c Department of Ophthalmology, Chungnam National University College of Medicine, Daejeon, Republic of Korea.

**Keywords:** acute optic neuropathy, case report, magnetic resonance imaging, optical coherence tomography, vincristine

## Abstract

**Rationale:**

A few cases of optic neuropathy presumed to be caused by vincristine have been reported. However, none described multimodal imaging findings. Here, we report abnormal magnetic resonance imaging (MRI) and optical coherence tomography (OCT) findings in a putative case of vincristine-induced optic neuropathy.

**Patient concerns:**

A 9-year-old boy with Burkett lymphoma who had had no visual problems noticed blurred vision in both eyes 22 days after the first maintenance therapy for lymphoma; the blurred gradually worsened. At that time, the best-corrected visual acuity was 20/200 and 20/100 in the right and left eyes, respectively.

**Diagnoses:**

Blood and imaging workup, and cerebrospinal fluid and genetic analyses, were performed; these included fundus photography, OCT, and MRI. We found no plausible cause of the optic neuropathy other than vincristine.

**Interventions:**

The scheduled chemotherapy was stopped, and the patient was managed with high-dose corticosteroids. However, as there was no improvement, plasma exchange was then performed.

**Outcomes:**

Three days after the initial examination, the visual acuity in both eyes was only light perception and projection. Signal intensity was abnormally high on 3-dimensional T2-weighted turbo spin echo and T2-weighted MRI images. Optic disc atrophy progressed to “total pallor”; thinning of the ganglion cell-inner plexiform and retinal nerve fiber layers also progressed. The patient was followed up for 7 months but showed no improvement in vision. There were no treatment-related complications.

**Lessons:**

We conclude that vincristine can cause optic neuropathy, and clinicians need to be alert to the possibility of optic neuropathy in any patient prescribed this agent.

OCT and MRI may help to diagnose optic neuropathy in pediatric patients. Periodic ophthalmologic examinations, including an OCT scan, may be useful.

## 1 Introduction

Vincristine is a mitotic inhibitor used to manage various pediatric tumors. Peripheral neuropathy is a well-known side effect of vincristine, and cranial nerve palsy is also seen occasionally.^[1–5]^ Other neurologic side effects are uncommon, with optic neuropathy being a particularly rare complication. Herein, we report a case of bilateral visual loss due to acute optic neuropathy in a male pediatric patient. We speculate that vincristine treatment was the underlying cause of the optic neuropathy.

## 2 Case report

The patient was a 9-year-old boy with Burkett lymphoma (stage IV, group C) managed with intravenous and intrathecal chemotherapy (Table 1). He had a normal diet, and there were no vision problems except for mild myopia. Three months previously, he was seen by an ophthalmologist; his vision was 20/20 in both eyes when wearing glasses.

**Table 1:** Chemotherapy scheduled of a patient with Burkett lymphoma (stage IV, group C).

2017–08–17∼08–24	Reduction phase (COP)
Cyclophosphamide, Vincristine (1 mg), Prednisone Intrathecal (MTX, Hydrocortisone, Cytarabine)	
2017–08–24∼09–18	Induction phase 1∼2 (COPADM)
Vincrisitin (2 mg), high-dose MTX, Cyclophosphamide, Doxorubicin, Prednisone Intrathecal (MTX, Hydrocortisone, Cytarabine)	
2017–10–11∼11–05	Consolidation 1∼2
High dose Ara-C, VP-16, Ara-C	
2017–12–4∼12–08	Maintenance 1
Vincrisitin (2 mg), high-dose MTX, Cyclophosphamide, Doxorubicin, Prednisone Intrathecal (MTX, Hydrocortisone, Cytarabine)	

**Figure 1:**
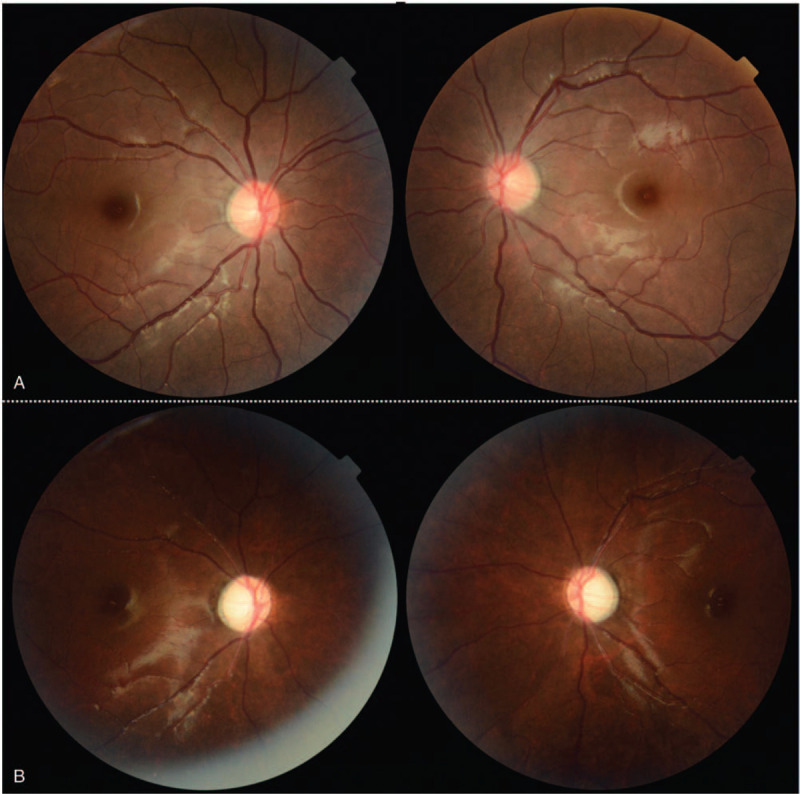
Fundus photographs of our patient. (A) There were no abnormal findings in the retina or optic disc at the initial examination. (B) Three months later, both optic discs showed “total pallor.” Left: right eye. Right: left eye.

**Figure 2:**
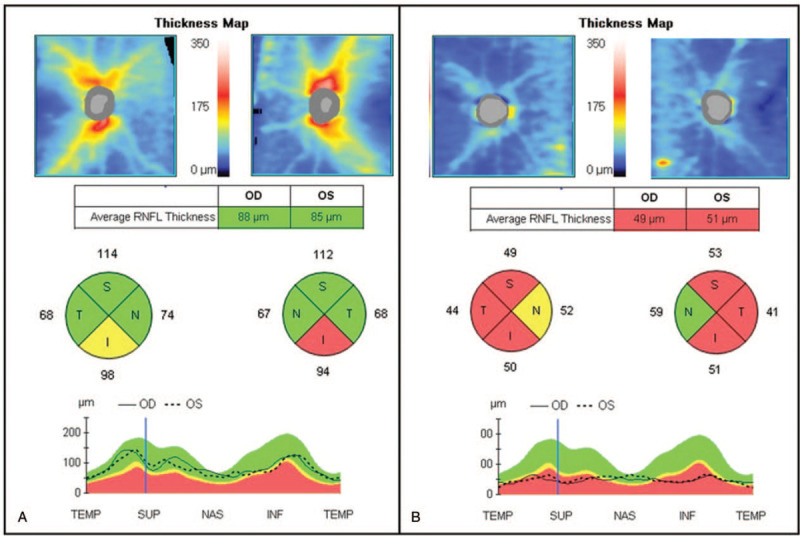
The thickness of the retinal nerve fiber layer (RNFL), as measured by spectral-domain optical coherence tomography (OCT). (A) The OCT scan performed at the initial examination showed borderline thinning of the bilateral RNFL. (B) Severe thinning of the RNFL was seen 3 months after the initial examination. I & INF = inferior sector, N & NAS = nasal sector, OD = right eye, OS = left eye, S & SUP = superior sector, T & TEMP = temporal sector.

**Figure 3:**
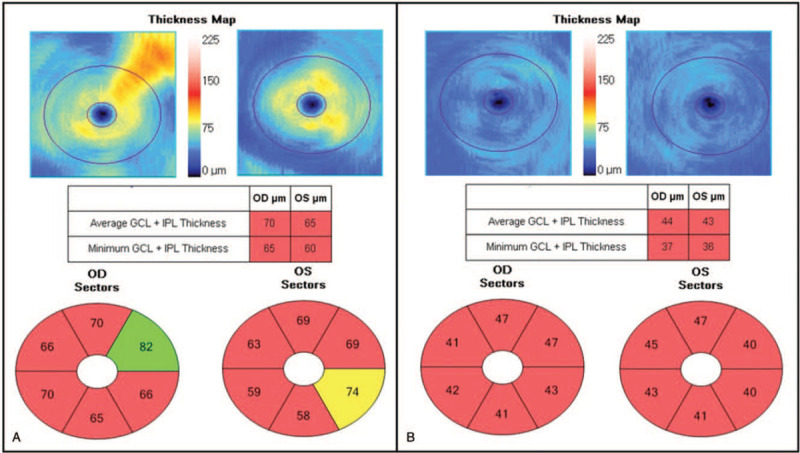
The thickness of the ganglion cell-inner plexiform layer (GC-IPL), as measured by spectral-domain optical coherence tomography. (A) The initial scan showed bilateral thinning of the GC-IPL. (B) Further thinning of the GC-IPL was seen 3 months after the initial examination. GCL + IPL = ganglion cell-inner plexiform layer, OD = right eye, OS = left eye.

**Figure 4:**
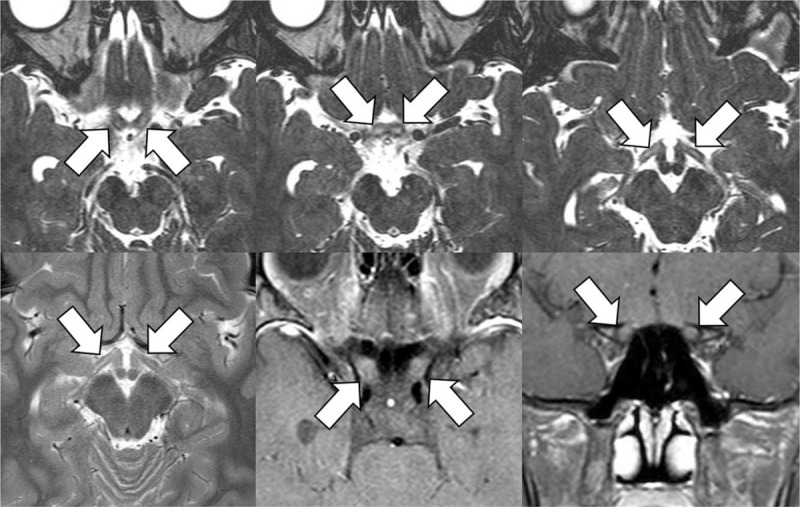
Magnetic resonance images of the patient. An axial 3-dimensional T2-weighted turbo spin echo image (top) showed hyperintensity in the bilateral intracranial segment of the optic nerves (top left, arrows), optic chiasm (top middle, arrows), and optic tracts (top right, arrows). An axial T2-weighted image (bottom left) showed hyperintensity in the bilateral optic tracts (arrows). Fat-suppressed contrast-enhanced axial (bottom middle) and coronal (bottom right) T1-weighted images showed mild enhancement in the bilateral intracranial segment of the optic nerves (arrows).

Twenty-two days after the first maintenance therapy, he noticed blurred vision in both eyes, which gradually worsened. He was seen by an ophthalmologist 2 days later; the best-corrected visual acuity was 20/200 and 20/100 in the right and left eyes, respectively. There was no ocular pain upon eye movement. The anterior segment and fundus were normal in both eyes (Fig. 1A). Optical coherence tomography (OCT; Cirrus HD OCT 5000 scanner; software version 10.0; Carl Zeiss Meditec, Dublin, CA) showed borderline thinning of the bilateral retinal nerve fiber layer and bilateral thinning of the ganglion cell-inner plexiform layer (GC–IPL; Figures 2A and 3A). The vision loss progressed despite the scheduled chemotherapy being stopped. Three days after the initial examination, the visual acuity in both eyes was only light perception and projection. Both pupils dilated moderately, and the light reflex was sluggish. Magnetic resonance imaging (MRI) showed abnormally high signal intensity in the bilateral intracranial optic nerves, optic chiasm, and optic tracts on axial 3-dimensional T2-weighted turbo spin echo images. T2-weighted images showed hyperintensity only in the bilateral optic tracts (Fig. 4). Blood workup indicated normal renal function, electrolytes, and liver function. Further blood tests, including of anti-neutrophil cytoplasmic antibody, angiotensin-converting enzyme, rapid plasma regain, vitamin B12, folate, and anti-aquaporin-4 antibody levels, were all normal, as was cerebrospinal fluid analysis. Genetic analysis did not reveal any of the four mutations seen in Leber hereditary optic neuropathy, and he had no history of exposure to other toxic substances.

The patient was managed using high-dose corticosteroids (intravenous methylprednisolone, 500 mg per day for 5 days); however, there was no improvement. Furthermore, there was no improvement in vision after four cycles of plasma exchange. Instead, light perception worsened and there was no light projection. Optic disc atrophy progressed to “total pallor”, and the thinning of the GC-IPL and retinal nerve fiber layer also progressed (Figs. 1B, 2B, and 3B). The patient was followed up for 7 months, during which time there was no change in his vision.

The high-dose corticosteroids and plasma exchange used as general treatment for idiopathic optic neuropathy had no complications.

Written informed consent for the publication of this case report was provided by the patient's parents, and the study was approved by the Ethics Committee of Chungnam National University Hospital, South Korea.

## 3 Discussion

Common side effects of vincristine include gastrointestinal disorder, lethargy, alopecia, and transient marrow suppression; it is also toxic to neural tissue, and reacts with microtubules and impairs axonal transport.^[6,7]^ Reversible peripheral neuropathy is the most common neurologic complication.^[3–5]^ Cranial nerve palsy, autonomic neuropathy, and cortical blindness have also been reported as neurologic complications.^[8]^

Optic neuropathy is a rare complication of vincristine use, with only a few cases reported. Norton and Stockman reported two pediatric patients who developed unilateral optic neuropathy approximately 1 week after being treated with vincristine.^[9]^ In that report, unlike our patient, both children were affected unilaterally and complained of pain; also, visual acuity improved after discontinuation of vincristine. Shurin et al^[10]^ reported bilateral optic atrophy in a 15-year-old girl that developed after 5 doses of weekly vincristine for management of medulloblastoma. Her vision improved after discontinuation of vincristine; however, optic disc pallor developed. Teichmann and Dabbagh^[11]^ reported severe bilateral optic neuropathy in a patient treated with vincristine for low-grade spinal cord astrocytoma. Weisfeld-Adams et al^[12]^ reported another case of optic neuropathy associated with vincristine: A 6-year-old boy presented with retro-orbital pain and rapid loss of vision 3 weeks after completing the sixth cycle of vincristine. There was no significant recovery of vision despite discontinuation of vincristine and administration of intravenous methyl-prednisolone, and fundoscopic examination revealed bilateral optic atrophy.

It is highly probable that vincristine caused the bilateral optic neuropathy in this case. There was no other obvious etiology, even after extensive workup and history-taking. However, vincristine was first administered about 4 months earlier than the onset of visual symptoms, although this temporal relationship is similar to that reported by Weisfeld-Adams et al.^[12]^ We hypothesize that chronic accumulation of toxic substances triggered a pathologic process.

This case is unique in several respects: vision decreased to light perception after only 5 days, and blindness ensued despite intensive medical care. These clinical features are more severe than any reported previously.

MRI findings were not described in most previous case reports, which were published decades ago such that modern MRI scanners were probably not available. Only one case, reported in 2007, had an MRI scan. However, there was no evidence of optic nerve swelling or optic pathway involvement.^[11]^ To our knowledge, the present case report of vincristine-induced optic neuropathy is the first to include abnormal MRI findings.

This case was characterized by an OCT finding that has not been reported before, namely substantial atrophy of the GC-IPL at the time of presentation. This suggests that an OCT scan might be useful for detecting early signs of optic neuropathy induced by vincristine before visual loss becomes obvious. This is important because most young children do not promptly report any decrease in vision, and OCT scans are noninvasive and easy to acquire. However, in accordance with the rarity of such cases, the fact that we reported only one is a clear limitation.

We suggest that clinicians need to be alert to the possibility of optic neuropathy in any patient receiving vincristine. Periodic ophthalmologic examinations, including an OCT scan, may facilitate early diagnosis. MRI findings may also aid diagnosis of this optic neuropathy.

## Author contributions

**Conceptualization:** Yeon-Hee Lee.

**Data curation:** Woo Hyuk Lee, Yeon-Hee Lee.

**Formal analysis:** Sun Kyoung You, Yeon-Hee Lee.

**Investigation:** Woo Hyuk Lee, Sun Kyoung You, Yeon-Hee Lee.

**Methodology:** Yeon-Hee Lee.

**Supervision:** Yeon-Hee Lee.

**Writing – original draft:** Woo Hyuk Lee.

**Writing – review & editing:** Woo Hyuk Lee, Yeon-Hee Lee.
